# Thai national guideline for nuclear medicine investigation in movement disorders: Nuclear medicine society of Thailand, the neurological society of Thailand, and Thai medical physicist society collaboration

**DOI:** 10.22038/AOJNMB.2023.75619.1531

**Published:** 2024

**Authors:** Tawika Kaewchur, Benjapa khiewvan, Wichana Chamroonrat, Praween Lolekha, Onanong Phokaewvarangkul, Tanyaluck Thientunyakit, Nantaporn Wongsurawat, Peerapon Kiatkittikul, Chanisa Chotipanich, Wen-Sheng Huang, Panya Pasawang, Tanawat Sontrapornpol, Nucharee Poon-iad, Sasithorn Amnuaywattakorn, Supatporn Tepmongkol

**Affiliations:** 1 Division of Nuclear Medicine, Department of Radiology, Faculty of Medicine, Chiang Mai University Thailand; 2 PET/CT and Cyclotron Center, Center for Medicine Excellence, Faculty of Medicine, Chiang Mai University Thailand; 3 Division of Nuclear Medicine, Department of Radiology, Faculty of Medicine Siriraj Hospital, Mahidol University, Bangkok Thailand; 4 Division of Nuclear Medicine, Department of Diagnostic and Therapeutic Radiology, Faculty of Medicine Ramathibodi Hospital, Mahidol University, Bangkok, Thailand; 5 Division of Neurology, Department of Internal Medicine, Faculty of Medicine, Thammasat University, Pathumthani Thailand; 6 Department of Medicine, Faculty of Medicine, Chulalongkorn University, Bangkok, Thailand; 7 Division of Nuclear Medicine, Department of Radiology, Faculty of Medicine Siriraj Hospital, Mahidol University, Bangkok, Thailand; 8 National Cyclotron and PET Centre, Chulabhorn Hospital, Chulabhorn Royal Academy, Bangkok, Thailand; 9 Department of Nuclear Medicine, Show Chwan Memorial Hospital, Changhua, 500 Taiwan (R.O.C.); 10 Division of Nuclear Medicine, Department of Radiology, King Chulalongkorn Memorial Hospital, Bangkok, Thailand; 11 Division of Nuclear Medicine, Department of Radiology, Faculty of Medicine, Chulalongkorn University, Bangkok, Thailand; 12 Chulalongkorn University Biomedical Imaging Group (CUBIG), Faculty of Medicine, Chulalongkorn University, Bangkok, Thailand; 13 Chula Neuroscience Center, King Chulalongkorn Memorial Hospital, Thai Red Cross Society, Bangkok, Thailand; 14 Center of Excellence in Cognitive Impairment and Dementia, Faculty of Medicine, Chulalongkorn University, Bangkok, Thailand

**Keywords:** Guidance, Procedure Standard, Nuclear Neurology, Parkinson

## Abstract

Movement disorders are chronic neurological syndromes with both treatable and non-treatable causes. The top causes of movement disorders are Parkinson's disease and related disorders. Functional imaging investigations with Single Photon Emission Computed Tomography (SPECT) and Positron Emission Tomography (PET) images play vital roles in diagnosis and differential diagnosis to guide disease management. Since there have been new advanced imaging technologies and radiopharmaceuticals development, there is a need for up-to-date consensus guidelines. Thus, the Nuclear Medicine Society of Thailand, the Neurological Society of Thailand, and the Thai Medical Physicist Society collaborated to establish the guideline for Nuclear Medicine investigations in movement disorder for practical use in patient care. We have extensively reviewed the current practice guidelines from other related societies and good quality papers as well as our own experience in Nuclear Medicine practice in movement disorders. We also adjust for the most suitability for application in Thailand and other developing countries.

## Introduction

 Movement disorders are the clinical syndrome of neurological conditions affecting speed, fluency, quality, and ability to control movement. Movement disorders could either be an excess of movement or a paucity of voluntary and involuntary movements unrelated to weakness or spasticity. Movement disorders are often induced by pathological changes within the brain, especially in the basal ganglia and its connection circuits. The cause of movement disorders may be unknown, although hereditary and environmental factors may play a role in some cases. In this national guideline for nuclear medicine, we focus on the clinical use of functional imaging with nuclear medicine techniques in idiopathic Parkinson’s disease (PD) and related disorders.

 PD is one of the most recognizable movement disorders of the central nervous system, mainly affecting the motor system. The prevalence of PD is approximately 1% of the population above 60 years and increases with age (1, 2). The diagnosis of PD is based on a neurological examination with cardinal motor features: bradykinesia, rigidity, rest tremor, and postural instability (3). Since many other disorders can mimic signs and symptoms like those of PD called Parkinsonism, the differential diagnosis with other forms of Parkinsonism can be challenging, especially early in the disease, when signs and symptoms of other Parkinsonism are not fully shown. A prospective clinicopathological study showed a diagnostic accuracy of 65% within five years of disease onset (4). A clear response to dopamine replacement therapy in an individual with PD could help to reinforce that an accurate diagnosis has been established. However, acute challenge testing with levodopa should not be used in the diagnosis of PD. Patients with suspected PD should be considered for a trial of chronic levodopa treatment (5). As the initial clinical diagnosis of PD is not fully precise, radiotracer neuroimaging could be an option for clinicians to improve diagnostic accuracy, which leads to better and more appropriate symptomatic management of patients, especially in the early stage of the disease (6). It has been well established that PD is associated with several non-motor symptoms. Some of these symptoms, such as hyposmia, constipation, depression, and REM sleep behavior disorder (RBD), might precede the onset of classical motor symptoms. Currently, there is no diagnostic test for PD. 

 Anatomical imaging, including Computed Tomography (CT) and Magnetic Resonance Imaging (MRI), is mainly used to exclude common secondary causes of Parkinsonism.

 A recent revision of the diagnostic criteria of PD has gained attention on functional images (7). 

 Although they are not directly implemented in the clinical diagnostic criteria for PD, they are an optional assessment to evaluate atypical Parkinsonism. The scintigraphic evidence of cardiac sympathetic denervation on radiolabelled metaiodobenzylguanidine (MIBG) scan and normal presynaptic dopaminergic imaging on Positron Emission Tomography (PET) or Single Photon Emission Computerized Tomography (SPECT) are included in the supportive and absolute exclusion diagnostic criteria for PD, respectively (7). Furthermore, functional neuroimaging is considered a marker of the preclinical or prodromal PD stage (7-9). In addition, it could be used in the differential diagnosis of PD from other parkinsonian syndromes (Table 1) and monitoring the PD progression in the research study.

**Table 1:** Causes of Parkinsonism

**Idiopathic Parkinson’s disease (PD)**	
**Secondary parkinsonism**	Drug-induced Vascular parkinsonism Normal-pressure hydrocephalus (NPH) Others; tumors, infection
**Atypical parkinsonism**	Progressive supranuclear palsy (PSP) Corticobasal degeneration (CBD) Multiple system atrophy (MSA) Dementia with Lewy bodies (DLB)
**Heredodegenerative parkinsonism**	

 Presynaptic dopaminergic imaging has been used to demonstrate dopaminergic dysfunction underlying PD symptoms. Among these imaging modalities, ^18^F-FDOPA PET and ^99m^Tc-TRODAT-1 SPECT are currently available in Thailand. Fluorodopa (FDOPA) PET is used to measure aromatic amino acid decarboxylase (AADC) activity and reflect the dopaminergic nerve terminal integrity. At the same time, ^99m^Tc-TRODAT-1 SPECT demonstrates the availability of presynaptic dopamine transporter (DAT). 

 However, the enzymatic / transporter targets visualized by FDOPA and TRODAT are often regulated by intrasynaptic dopamine concentration, and they can under- or over-estimate dopaminergic terminal densities. In PD, both techniques show a characteristic pattern of asymmetric reduction in striatal uptake that affects the posterior putamen contralateral to the clinically affected limb with a relatively preserved head of caudate (10-12). The reductions in tracer uptake also correlate with nigral dopamine cell counts and the degree of motor dysfunction, especially with bradykinesia. The findings mentioned above can also be found in atypical Parkinsonism. Thus, they cannot reliably differentiated among PD and others (13-15).

 Regional cerebral glucose metabolism by fluorodeoxyglucose (^18^F-FDG) PET reflects neuronal activity. Therefore, in the case of degenerative Parkinsonism, FDG PET might distinguish iPD patients from those with atypical Parkinsonism, such as multiple system atrophy (MSA), progressive supranuclear palsy (PSP), corticobasal degeneration (CBD), and dementia with Lewy bodies (DLB) (10, 11). 

 Also, the cortical glucose hypometabolism in parkinsonian patients might suggest cortical Lewy body dementia, such as Parkinson’s disease dementia (PDD) and DLB.

 Myocardial innervation scintigraphy with radio-labeled metaiodobenzylguanidine (MIBG) is a marker of the integrity and distribution of the postganglionic sympathetic nerve terminals. 

 Thus, in PD, the cardiac sympathetic denervation, as shown by the reduction or absence of MIBG uptake, can be used as a biomarker in the supportive criteria for diagnosing PD and differential diagnosis from other atypical Parkinsonism (12, 16).

 To effectively use functional imaging with nuclear medicine, all members, including physicians, neurologists, radiologists, nuclear medicine professionals, and technicians, should have consensus information on these imaging techniques. In addition, functional imaging with nuclear medicine in movement disorders should be considered an aid to clinical diagnosis in patients with uncertain diagnoses. However, there are limitations to accessing PET/CT services in some regions of Thailand. We thus propose alternative brain SPECT studies for each purpose.

***Parkinsonism***

 Parkinsonism is a clinical syndrome characterized by the following cardinal features: rigidity, bradykinesia and rest tremor, typically asymmetrically affecting an upper extremity at onset (17). Postural instability is uncommon at the onset of PD but usually develops within several years of disease progression. Parkinsonism is a term used to describe movement symptoms associated with several conditions, including Idiopathic Parkinson’s disease, atypical Parkinsonism (Parkinsonism Plus), and secondary Parkinsonism. Differences in abnormal protein are related to degenerative Parkinsonism and represented by various clinical parkinsonian syndromes such as synucleinopathies (PD, DLB, MSA) and tauopathies (PSP and CBD). The most reliable clinical diagnosis of PD is made with typical symptoms and signs (above) together with significant improvement with dopamine replacement medical therapy.

***Idiopathic Parkinson’s disease (PD)***

 In general, PD can be divided according to their manifestations into Prodromal and Clinical PD stages.

***Prodromal Parkinson’s disease***

 Prodromal-PD refers to the stage at which individuals have symptoms and signs that indicate a higher than average risk of developing PD (such as hyposmia, constipation, and REM sleep disorder), but do not manifest Parkinsonism sufficient to diagnose PD according to the diagnostic criteria (7). 

***Clinical Parkinson’s disease***

 Clinical PD refers to the stage at which individuals present classical motor signs (bradykinesia, rigidity, and rest tremor) of PD and are diagnosed as PD according to the diagnosis criteria (3, 7, 8). 

***Parkinson’s Disease Dementia (PDD)***

 Parkinson’s disease dementia refers to the stage at which individuals with PD, who are diagnosed with PD according to established criteria, present with insidious onset and slow progression of cognitive impairment or dementia syndromes (18).

***Atypical Parkinsonism***

 Atypical Parkinsonism previously known as a Parkinsonism-plus syndrome is a sporadic entity of neurodegenerative parkinsonian syndromes that could be distinguished from Parkinson's disease by a relative lack of response to dopamine replacement therapy and their (eventual) additional or “plus” features including, vertical supranuclear palsy (PSP), autonomic dysfunctions (MSA), dystonia or myoclonus or cerebral cortical signs (CBD), and early cognitive impairment (DLB) (19). 

 Therefore, it is necessary to identify red flag signs that should not be found in typical PD, and these signs support the diagnosis of atypical Parkinsonism.

***Progressive Supranuclear Palsy (PSP)***

 In contrast to PD, PSP or Steele- Richardson-Olszewski syndrome presents a symmetrical akinetic–rigid syndrome. Rigidity in PSP usually predominantly involves the trunk and neck, causing early postural and gait instability with falls (20). Therefore, any elderly patient with truncal or neck rigidity with minimal limb involvement and impaired postural reflexes should be suspected of PSP. Vertical supranuclear gaze palsy is a pathognomonic sign of PSP, especially in a downward position (20). 

 Impairment of frontal executive functions is frequent in PSP, and dementia, often of a frontal type, is common in later diseases leading to memory impairment, expressive dysphasia, and limb apraxia.

***Multiple System Atrophy (MSA)***

 MSA has two subtypes: predominant Parkinsonism (MSA-P, 60%) or predominant cerebellar symptoms (MSA-C, 40%). In addition to motor impairment, autonomic dysfunctions are a compulsory component of MSA-especially urinary incontinence, erectile dysfunction, or orthostatic hypotension (21). 

 MSA-P presents with a hypokinetic-rigid parkinsonian syndrome that tends to be more symmetrical and less responsive to levodopa than PD. In contrast, MSA-C usually presents with signs of cerebellar dysfunction including, limb and gait ataxia, scanning dysarthria, and nystagmus. The diagnostic criteria for MSA require at least one symptom of autonomic dysfunction (orthostatic hypotension, urinary incontinence, erectile dysfunction) after excluding alternative symptomatic causes (21). 

***Corticobasal Degeneration (CBD)***

 CBD is progressive atypical Parkinsonism that results from neurodegeneration of the basal ganglia and the cerebral cortex. The combination of hypokinetic-rigid parkinsonian syndrome with additional signs, including dystonia, myoclonus, and cortical symptoms, is the characteristic feature of CBD, and is typically resistant to levodopa therapy (19). 

 Most patients eventually develop progressive immobility and cognitive impairment in a couple of years after diagnosis.

***Dementia with Lewy Bodies (DLB)***

 Dementia with Lewy Bodies refers to a dementia syndrome with underlying cortical Lewy-related pathology that precedes parkinsonian motor signs or begins within one year from its onset (22). The definition of DLB is a progressive cognitive decline of sufficient magnitude to interfere with normal social or occupational functions or with usual daily activities. Prominent or persistent memory impairment may not necessarily occur early but is usually evident with progression (22). Deficits on attention, executive function tests, and visuoperceptual ability may be especially prominent and occur early (22). In addition, four clinical core features are identified and usually occur and may persist throughout the course including, 1) fluctuating cognition with pronounced variations in attention and alertness, 2) recurrent visual hallucinations that are typically well formed and detailed, and 3) REM sleep behavior disorder, which may precede cognitive decline and 4) one or more spontaneous cardinal features of Parkinsonism.

***Drug-Induced Parkinsonism (DIP)***

 Drug-induced Parkinsonism is the most common movement disorder induced by drugs that affect dopamine receptors (23). Since the clinical manifestations of DIP are very similar to those of Parkinson’s disease (PD), patients with DIP are frequently misdiagnosed as having PD. 

 Typical antipsychotics, also known as neuroleptics, are the most common causes of DIP. In addition to antipsychotics, gastro-intestinal (GI) motility drugs, calcium channel blockers (CCBs), and antiepileptic drugs have been found to induce DIP. Dopamine transporter (DAT) imaging may be used to diagnose various etiologies of Parkinsonism, including DIP (24).

***Scans Without Evidence of Dopaminergic Deficit (SWEDDs)***

 SWEDDs are a heterogeneous group of patients who fulfill clinical diagnostic criteria for PD, but have normal dopaminergic functional imaging (25). Therefore, alternative diagnoses of SWEDDs have been suggested include essential tremor (ET), depression, vascular or psychogenic Parkinsonism, dopa-responsive dystonia, and rare causes of supranigral Parkinsonism.

**Guideline methodology and panelists**

**Figure 1:**
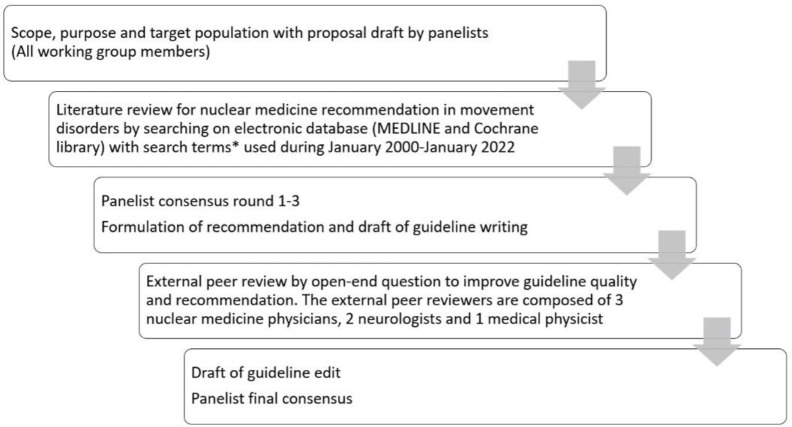
Guideline development process

 A panel of specialists in nuclear medicine, medical physicists and technologists as well as neurologists was established for guideline development. The involving panelists are listed in table 2. The guideline development process was shown in figure 1.

**Table 2:** Panelist composition

**Name**	**Field of expertise**	**Institution**	**Country**
Supatporn Tepmongkol	Nuclear Medicine	Chulalongkorn University	Thailand
Tawika Kaewchur	Nuclear Medicine	Chiang Mai University	Thailand
Benjapa Khiewvan	Nuclear Medicine	Mahidol University	Thailand
Wichana Chamroonrat	Nuclear Medicine	Mahidol University	Thailand
Tanyalak Thientunyakit	Nuclear Medicine	Mahidol University	Thailand
Nantaporn Wongsurawat	Nuclear Medicine	Khon Kaen University	Thailand
Peerapon Kiatkittikul	Nuclear Medicine	National Cyclotron and PET Centre	Thailand
Chanisa Chotipanich	Nuclear Medicine	National Cyclotron and PET Centre	Thailand
Wen-Sheng Huang	Nuclear Medicine	Taipei Medical University Hospital	Taiwan
Panya Pasawan	Medical Physicist and Technologist	Chulalongkorn University	Thailand
Tanawat Sontrapornpol	Medical Physicist and Technologist	Chulalongkorn University	Thailand
Nucharee Poon-iad	Medical Physicist and Technologist	Mahidol University	Thailand
Sasithorn Amnuaywattakorn	Medical Physicist and Technologist	Mahidol University	Thailand
Praween Lolekha	Neurologist	Thammasat University	Thailand
Onanong Phokaewwarangkul	Neurologist	Chulalongkorn University	Thailand

Note:

1. Search terms are Parkinsonism, Parkinson’s disease, scintigraphy, SPECT, PET, diagnosis, differential diagnosis, and progression

2. All aforementioned search terms were used for keyword and mapping with medical subject headings in the hierarchy of central nervous system and movement disorders

3. Example of search formula;

Concept 1: Parkinsonism OR possible synonyms (eg. Parkinson plus syndrome, corticobasal degeneration)

Concept 2: Scintigraphy OR SPECT OR PET

Concept 3: Diagnosis OR differential diagnosis OR progression

Then, searches these combinations to find all articles that include a search term from each concept: Concept 1 AND Concept 2 AND Concept 3

## Objectives

1. To identify the role of nuclear medicine studies in patients with Parkinsonism.

2. To assist referrers in requesting the most appropriate procedure for Parkinsonism patients.

3. To identify scientific evidence that is useful to assist nuclear medicine professionals in recommending, performing, interpreting, and reporting the results of nuclear medicine investigations in Parkinsonism patients.

## Indications and Contraindications

***Indications for Nuclear Medicine Imaging in Parkinsonism with the Evidence Base and Flow of Investigation***

 The indication for nuclear medicine imaging in Parkinsonism was reviewed base on the established criteria for rating diagnostic accuracy studies and recommendations (American Academy of Neurology, 2017). The evidence-based reviews have used a standard method using literature searches performed using electronic databases (Medline, Cochrane Library) with search keywords: parkinsonism, Parkinson's disease, scintigraphy, SPECT, PET, diagnosis, progression, differential diagnosis, from January 2006 to June 2022. The included studies have to be human-based with full English articles. Each study was rated by 2 independent panelists (P.L. and O.P.). The overall recommendation for clinical practice was then assessed and classified as A: established effective, B: probably effective, C: possible effective, and U: data inadequate.

**Figure 2:**
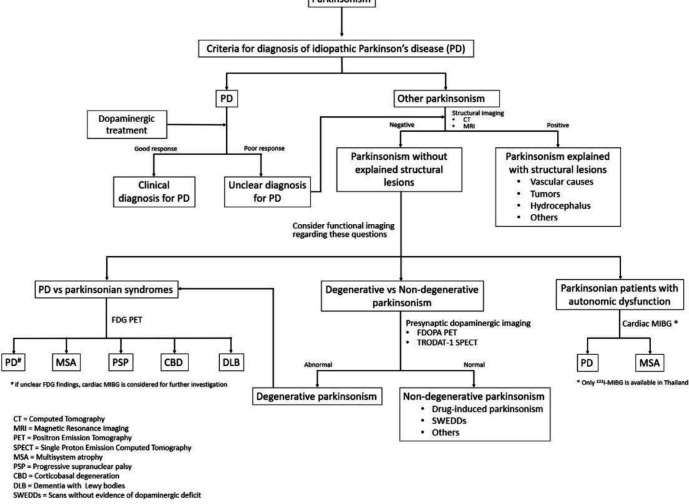
Flow of Investigation in Movement Disorder

 The evidence of nuclear medicine investigation in Parkinsonism is shown in table 3 and the flow of imaging investigation in movement disorder is shown in Figure 2.

***Contraindications***

- Pregnancy
- Breastfeeding

**Table 3:** Level of Recommendation for Nuclear Medicine Imaging in Parkinsonism

	**Level of Recommendation**
**Support a diagnosis of PD**	
FDOPA PET	B
DAT SPECT/PET	A
FDG PET	U
Cardiac MIBG	U
**Assessment of the progression of PD**	
FDOPA PET	B
DAT SPECT/PET	B
FDG PET	U
Cardiac MIBG	U
**Differentiate PD from atypical parkinsonism**	
FDOPA PET	U
DAT SPECT/PET	U
FDG PET	C
Cardiac MIBG	U

**A**: established effective, **B**: probably effective, **C**: possibly effective, and **U**: data inadequate

## Procedure

***Patient Preparation***

***I. TRODAT-1 SPECT***

 Patient Preparation and Precautions (26)

Check for medications on table 4 that may alter tracer binding and need to withdraw (if possible) for at least five half-lives.

**Table 4:** Lists of Medication that May Alter TRODAT-1 Uptake

**Medication**	**Effect of the medication**
**Cocaine**	Severely decrease binding to DaT
**Amphetamines**	
d-amphetamine methamphetamine methylphenidate	Decrease striatal binding
**Central ** **nervous system stimulants **	
ephedrine phentermine *particularly when used as tablets	Decrease striatal binding
**Modafinil**	Decrease striatal binding
**Some antidepressants** (mazindol, bupropion, radafaxine)	Decrease striatal binding
**Adrenergic agonists**	
phenylephrine norepinephrine	Increase striatal binding
**Anticholinergic drugs**	
benztropine	Decrease striatal binding ratios
other anticholinergics	Might increase these ratios, which will likely not affect visual assessments
**Opioids **(only fentanyl)	Decrease striatal binding
**Bupropion, fentanyl, and some anesthetics** (ketamine, phencyclidine, and isoflurane)	May decrease binding to DaT
**Selective serotonin reuptake inhibitors**	May increase binding to DaT somewhat but should not interfere with visual interpretation
**Cholinesterase inhibitors and neuroleptics**	Probably do not interfere significantly with binding to DaT
**Anti-Parkinsonian drugs ** (e.g., L-dihydroxyphenylalanine, dopamine agonists, monoamine oxidase B inhibitors, N-methyl-D-aspartate receptor blockers, and catechol-O-methyltransferase inhibitors in standard dosages)	Do not interfere with binding to DaT to any significant degree

***II. Cardiac MIBG Imaging***

Patient Preparation and Precautions (27)

1. Withdrawal of medications listed in table 5, which may alter tracer uptake, is advised. In the case of hypertension, the recommended anti-hypertensive medications are shown in Table 6.
2. Thyroid Blockade

The thyroid blockade agent must be given one day before tracer injection and continue for one to two days for ^123^I-MIBG or two to three days for ^131^I-MIBG (27). The recommendations for

thyroid blockade are shown in table 7.

On the Acquisition Day

1. Evaluate baseline clinical, vital signs, and general physical examination.

2. Slowly inject MIBG and observe pallor, vomiting, or fainting.

3. Encourage patients to drink a large volume of fluid and void before the study. 

Monitor vital signs, and observe the adverse reactions of MIBG (tachycardia, pallor, vomiting, abdominal pain).

**Table 5:** Medication and drugs of abuse interfering I-131 MIBG uptake

**Medication**	**Approved name**	**Effect of the medication**	**Withdraw period recommendation in tumor**
**I. Cardiovascular and sympathomimetic drugs**			
Antiarrhythmics for ventricular arrhythmias	Amiodarone	Inhibition uptake and depletion	Not practical to withdraw
Combined α/β-blocker	Labetalol*	Inhibition uptake and depletion	72 hours 21 days*
Adrenergic neuron blocker	Bretylium*, Guanethidine*, Reserpine*	Depletion and transport inhibition	48 hours 14 days*
α-Blocker	Phenoxybenzamine (intravenous doses only)	Unknown mechanisms	15 days
Calcium channel blockers	Diltiazem, Nifedipine*, Nimodipine	Increased uptake and retention	24 hours 14 days*
	Amlodipine*, Felodipine, Isradipine, Lacidipine, Lercanidipine, Nicardipine*, Nisoldipine, Verapamil	Increased uptake and retention	48 hours 14 days*
Inotropic sympathomimetics	Dobutamine, Dopamine, Dopexamine	Depletion of granules	24 hours
Vasoconstrictor sympathomimetics	Ephedrine	Uptake inhibition	24 hours
	Metaraminol, Norepinephrine, Phenylephrine	Depletion of granules	24 hours
β2 stimulants (sympathomimetics)	Salbutamol, Terbutaline, Eformoterol, Bambuterol, Fenoterol and Salmeterol	Depletion of granules	24 hours
Other adrenoreceptor stimulants	Orciprenaline	Depletion of granules	24 hours
Systemic and local nasal decongestants, compound cough and cold preparations	Ephedrine	Uptake inhibition	24 hours
	Xylometazoline, Oxymetazoline	Depletion of granules	24 hours
	Pseudoephedrine, Phenylephrine	Depletion of granules	48 hours
Sympathomimetics	Brimonidine, Dipivefrine, Phenylpropanolamine*, Ephedrine*, Pseudoephedrine*, Phenylephrine*, Amphetamine*, Dopamine, * Isoproterenol*, Salbutamol*, Terbutaline*, Phenoterol*, Xylometazoline*	Depletion of granules	48 hours 7-14 days*
**II. Neurological drugs**			
Antipsychotics (neuroleptics)	Chlorpromazine*, Perphenazine, Prochlorperazine, Promazine, Thioridazine	Uptake inhibition	24 hours 21-28 days*
	Fluphenazine*	Uptake inhibition	24 hours, or 1 month for depot 21-28 days*
	Benperidol, Pericyazine, Sulpiride, Trifluoperazine, Quetiapine	Uptake inhibition	48 hours
	Droperidal*, Flupentixol, Haloperidol*, Zuclopenthixol	Uptake inhibition	48 hours, or 1 month for depot 21-28 days*
	Levomepromazine, Pimozide, Amisulpride	Uptake inhibition	72 hours
	Zotepine	Uptake inhibition	5 days
	Risperidone	Uptake inhibition	5 days or 1 month for depot
	Clozapine	Uptake inhibition	7 days
	Olanzapine	Uptake inhibition	7 -10 days
	Sertindole	Uptake inhibition	15 days
	Pipotiazine	Uptake inhibition	1 month for depot
	Lozapine*	Uptake inhibition	21-28 days*
Sedating antihistamines	Promethazine*	Uptake inhibition	24 hours 21-28 days*
Opioid analgesics*	Tramadol*	Uptake inhibition	24 hours 7–14 days*
Tricyclic antidepressants	Clomipramine, Dosulepin (dothiepin), Doxepin*, Imipramine*, Nortriptyline	Uptake inhibition	24 hours 7–14 days*
	Amitriptyline*, Amoxapine*, Lofepramine, Trimipramine	Uptake inhibition	48 hours 7–14 days*
Tricyclic-related antidepressants	Maprotiline*, Mianserin, Trazolone*, Venlaflaxine	Uptake inhibition	48 hours 21-28 days*
	Reboxetine	Uptake inhibition	72 hours
	Mirtazepine	Uptake inhibition	8 days
CNS stimulants	Cocaine*, Caffeine	Uptake inhibition	24 hours 7-14 days*
	Amphetamines (e.g. dexamfetamine), Methylphenidate	Depletion of granules	48 hours
	Modafinil	Unknown mechanisms	72 hours
	Atomoxetine	Uptake inhibition	5 days

NOTE:

Modified from;

EANM guideline >>> 131I-/123I-Metaiodobenzylguanidine (MIBG) scintigraphy: procedure guidelines for tumor imaging; Published online: 20 July 2010, EANM 20103

* Cardiac MIBG guideline and study review4. Withdrawal period is preferred using the time interval for cardiac MIBG studyHowever, if it cannot be applied, using tumor recommendation withdrawal period is also acceptable

**Table 6:** Recommended Antihypertensive Drugs for 131I-MIBG Study

**Medication**	**Example of Medications**
α-blocker	Selective α1-blockers: Prazosin, Terazosin, Doxazosin
	Phenoxybenzamine
β-blocker	Propranolol

**Table 7:** Thyroid Blockade in Adults

Potassium iodate	170 mg
Potassium iodide	130 mg
Lugol’s 1%	1 drop/kg to a maximum of 40 drops (20 drops twice a day)
Potassium perchlorate	400 mg

***III.*** ***FDG PET***

Patient Preparation and Precautions (28-30)

1. Patients should fast for at least four hours to allow optimal cerebral FDG uptake.
2. Advise the patients to avoid excessive stimulants which may affect brain metabolism at least 24 hours.

- Food or drinks such as caffeine, cola, alcohol, and energy drinks.

- Smoking and excessive exercise.

3. Control blood glucose in diabetic patients

4. Recommended medications to be withdrawn are shown in table 8.

*On Acquisition Day*

1. Blood glucose must be checked in every case. The optimal blood glucose level should not exceed 160 mg/dl (30). However, high blood glucose level exceeding 160 mg/dl is not a contraindication but needs a caution to interpret due to elevated brain background activity.

2. Adequate-hydration is advised.

3. Check the patient’s ability to lie still during the whole acquisition time.

4. Intravenous catheterization should be placed at least ten minutes before injection.

5. Place the patient in a quiet, dimly lit room with open eyes and unplugged ears with minimal to no movement and no external stimulus for at least 30 minutes before and after tracer injection.

6. The optimal uptake time is 30-60 minutes.

7. Instruct the patient to void before scanning for patient comfort and to minimize radiation exposure to the bladder.

8. In uncooperative patients, sedation (28) can be applied and should be given just before image acquisition. 

9. Continually monitor vital signs, including pulse oximetry, during the entire scanning procedure, especially in those receiving sedation. Emergency backup should be available.

**Table 8:** Medications interfering FDG uptake

**Medication**	**Duration of withdrawal**
**Amphetamine**	4-6 half-life
**Cocaine**	4-6 half-life
**Anesthetics**	
Propofol	4-6 half-life
Isoflurane	
Barbiturate	
**Benzodiazepine**	4-6 half-life
**Corticosteroid**	4-6 half-life
**Neuroleptics (anti-psychotics)**	
Haloperidol	4-6 half-life
Chlorpromazine	
**Cholinesterase inhibitor**	
Donepezil	4-6 half-life
Rivastigmine	

***IV. FDOPA PET***

Patient Preparation and Precautions (31)

1. Patients should fast for at least two to six hours. Do not take any amino-acid-containing foods six hours before the procedure.

2. Medication withdrawal: stop COMT inhibitors for five half-lives before scanning. Other drugs used for PD can be continued. 

*On Acquisition Day*

1. Pre-medication: Pure carbidopa 2 mg/kg (max 150 mg) orally, 90 minutes prior to ^18^F-FDOPA injection is optional (32).

2. Pre-hydration: drink up to one liter of water before intravenous administering ^18^F-FDOPA (200 MBq) to decrease the ^18^F-FDOPA urinary concentration.

3. Conscious sedation (28) (e.g., a short-acting benzodiazepine such as intravenous midazolam) can be applied in uncooperative or uncontrolled-symptom patients. Sedation should be given at least 20 minutes after radiopharmaceutical administration.

4. Continually monitor vital signs, including pulse oximetry, during the entire scanning 

procedure, especially in those receiving sedation. Emergency backup should be available.

***Radiopharmaceuticals and radiation dosimetry***

 Radiopharmaceutical dosage is shown in Table 9 (28, 33-36), and their characteristics are shown on table 10.

***Radiation Dosimetry***

 Radiation dosimetry in SPECT and PET studies is shown in Table 11 (29, 33, 35, 37).

## SPECT Imaging Acquisition and Data Processing

***TRODAT-1 Brain SPECT***

 The patient positioning, image acquisition and data processing of TRODAT-1 brain SPECT are shown in Table 12 (38).

**Table 9:** Radiopharmaceutical Dosages

**Radiopharmaceuticals**	**Dosage**
^99m^Tc-TRODAT-1	740 MBq (20 mCi)
^131^I-MIBG	111 MBq (3 mCi)
^18^F-FDOPA	3.5 MBq/kg (0.09 mCi/kg)
^18^F-FDG	185-370 MBq (5-10 mCi)

Note: Radiopharmaceuticals dose in this table is general recommendation. Dose adjustment should be considered for local regulatory approval as well as types of scanners used

**Table 10:** Radiopharmaceuticals Characteristics

**Agent​**	**Peak brain activity(min)​**	**Brain uptake​** **(%** **of injected dose)​**	**Brain/Bg​ Ratio​**	**GM: WM​ ratio​**	**Imaging time​**	**Brain washout​**
TRODAT-1	30-40 (striatum in baboon*)	<2	2:1 (basal ganglia: Occipital) 1.5-2.2:1 (Striatum: Cerebellum)	N/A	3.5-5 hours	Slow washout from the striatum after 90 mins
FDOPA	30-50​	6​	Higher​	2.5-4.1:1​	>30 min​	none​
**Agent**	**Peak cardiac activity** **(min)​**	**Cardiac** **uptake** **(%)**	**Early heart /mediastinum**	**Delayed heart /mediastinum** **ratio** **(4 hrs)**	**Imaging time**	**Cardiac washout**
MIBG	120	< 1% at less than 24 hours	2.41 + 0.26	2.66 + 0.47	15-30 minutes for early 3-4 hours for delayed images	Yes

**Table 11:** Radiation Dosimetry in SPECT and PET Studies

**Radiopharmaceuticals**	**Dose in MBq unit (mCi)**	**Organ receiving the highest dose mGy/MBq**	**Effective dose mSv/MBq**
^99m^Tc-TRODAT-1	740 (20)	0.047 (liver)	0.015
^131^I-MIBG	44.4 – 81.4 (1.2 – 2.2)	0.83 (liver)	0.14
^18^F-DOPA	200 (5.4)	0.15 (Bladder wall)	0.0199
^18^F-FDG	185-740 (5-20)	0.13 (bladder wall)	0.019

Note: Low-dose CT scan is used for attenuation correction. The effective dose of a low dose CT scan is about 20 µSv and 220-450 µSv in a high dose or diagnostic CT

**Table 12:** TRODAT-1 SPECT Acquisition and Data Processing

**Patient Positioning**	
Supine and arms down position on the scanner table	
Immobilization to maintain head position and reduce movement	
Patient’s head in the center of the field of view	
The head held straight (not tilted left or right) with the line from ear to the eye (the canthomeatal line) perpendicular to the detector surface, if possible	
Use the smallest radius of rotation as possible or automated contour setting from patient to detector for imaging to ensure maximum image resolution	
**Data Acquisition Parameters**	
Uptake time	4 hours
Instrument	SPECT
Collimator	Fan beam or Parallel hole (LEHR/LEUHR)*
Energy setting	140 keV, 15-20% energy window
Nuclide	^99m^Tc
Matrix size	≥ 128×128
Zoom	To gain at least pixel size smaller than 1/3 to 1/2 of the expected resolution
Scan mode	Step and shoot or continuous
Rotation per view	≤ 3° (total of 360° rotation)
Time per view	About 15-30 sec/projection (minimum total count 5 x 106 counts)
Scatter correction	Optional, but recommended
Reconstruction	3D-OSEM/FBP **
Slice thickness	Possible 3-5 mm (for maximal pixel resolution)
**If SPECT/CT (CT for attenuation correction)**	
CT voltage	120-140 kV
CT current	30-80 mA (depends on vender’s recommendation)
Slice thickness	Same as SPECT slice thickness for AC
**Data Processing **	
Review projection data in cine mode and sonogram for an initial determination of image quality, patient motion, and artifacts	
Reconstruct by OSEM or FBP algorithm	
Select resolution recovery filter, i.e., Metz filter or type of low pass filter, i.e., Butterworth, Hamming, or Hanning	
Optimize reconstructing parameters, i.e., cut-off, order, iterations, and subset, depending on the injected activity, camera sensitivity, and resolution	
Apply attenuation correction using either calculated (e.g., Chang’s method) or measured (e.g., Gadolinium source or CT scan) attenuation	
Apply the scatter correction (optional) to improve the image signal-to-noise ratio with various methods. The most popular one is the triple-energy window correction.	

Note:

*Low Energy High Resolution (LEHR) or Low Energy Ultra High Resolution (LEUHR)

** Ordered Subsets Expectation Maximization (OSEM) or Filtered Back Projection (FBP)

***Cardiac MIBG Planar Imaging***

 The patient positioning, image acquisition and data processing of cardiac MIBG planar image are shown in Table 13.

***Brain PET Imaging Acquisition and Data Processing***

 The patient positioning, image acquisition and data processing of FDG brain and FDOPA brain PET are shown in Table 14.

**Table 13:** Cardiac MIBG Planar Image Acquisition and Data Processing

**Patient Positioning**	
Comfortably supine and arms down position on the imaging table	
Immobilization to maintain chest position and reduce movement	
The patient’s heart is in the center of the field of view, covering the mediastinum and whole lung	
**Data Acquisition Parameters**	
Uptake time	Eary image at 15-30 minutes post tracer injection
	Delayed image at 3- 4 hours post tracer injection
Instrument	SPECT
Collimator	High Energy
Energy setting	364 keV, 15-20% energy window
Nuclide	^131^I
Matrix	256×256
Mode	Static
View	Anterior
Zoom	1.67 - 2 ( 〈 FOV)
Scan time	5 – 10 min
**Data Processing**	


**Table 14:** FDG Brain and FDOPA Brain PET Acquisition and Data Processing

**Patient Positioning**	
Comfortably supine and arms down position on the imaging table	
Place the patient’s head in a holder using foam or folded sheet so that the canthomeatal line is in a vertical orientation	
Immobilization to maintain head position and reduce movement	
Patient’s head in the center of the field of view	
**Data Acquisition Parameters**	
Uptake time	30-60 minutes for FDG PET
	90 minutes for FDOPA PET
**CT**	
Scan type	Helical
Rotation time	0.75-1 section
Matrix	512
Slice thickness	3-5 mm
Slice increments	Continuous
Pitch	≤ 1
CT voltage	120-140 kV
CT current time	≤ 50 mAs (Low dose CT)
	≤ 250 mAs (Diagnostic CT)
**PET**	
Energy setting	511 keV, 15-20% energy window
Nuclide	^18^F
Mode	2D, 3D in list mode
Scan direction	Towards head
Scan duration	5-15 minutes for FDG PET (vary depending on vendor’s recommendation)
	30 minutes for static FDOPA PET image (may vary depending on vendor’s recommendation) Dynamic FDOPA PET image is optional with 5 minutes/ frame, a total of 18 frames
CT Attenuation correction	Yes
Scatter correction	Yes
Reconstruction	Iterative (OSEM) or equivalent
**Data Processing **	
Preview image for patient motion and ensure PET and CT images are matched before performing CT attenuation correction	
Images are reconstructed in the transaxial plane of at least 128X128 matrix size, zoom 2	
The typical pixel size is 2-4 mm	
Depending on the resolution of the PET system, a final image resolution may vary between 2.5-10 mm. FWHM. This FWHM typically yields adequate image resolution and signal-to-noise ratios	
The reconstruction parameters can be varied. Please refer to the manufacturer’s recommendations for the best choices of iterations, subsets, and smoothness.	

## Interpretation

***Cardiac MIBG***

***General Considerations***

 The radiolabeling of MIBG with iodine-123 or iodine-131 enables the visualization and quantification of cardiac postganglionic sympathetic innervation in vivo. Cardiac MIBG scintigraphy has demonstrated its capability to differentiate between PD and atypical Parkinsonism, essential tremor (ET), and normal control (33, 39-43). Furthermore, it can also differentiate between dementia with Lewy bodies and other types of dementia (43). In Thailand, only the ^131^I isotope is available. Thus, ^131^I-MIBG is used in this guideline.

***Image Data Display***

**Figure 3:**
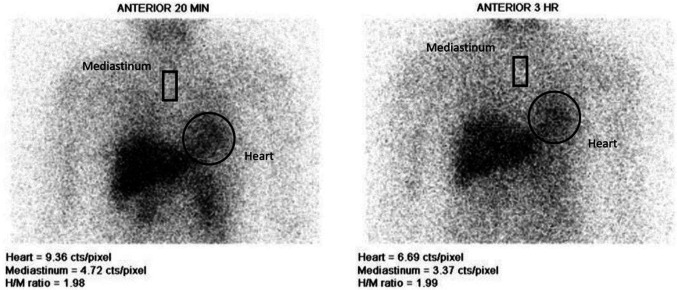
I-131 MIBG anterior view of the chest at 20 minutes and three hours. ROIs were drawn around the heart and mediastinum to calculate the H/M ratio

 The anterior view of chest planar imaging is displayed in grayscale at 15-30 minutes (early scan) and 3-4 hours (delayed scan). The ratio of ^131^I-MIBG uptake in the heart to the mediastinum (H/M ratio) is quantified by drawing the region of interest (ROI) around the heart and the upper mediastinum, as shown in Figure 3.

***Image Interpretation***

 Semi-quantitative analysis using heart/ mediastinum (H/M) ratio is often used for cardiac ^131^I-MIBG analysis. The mediastinum is often used as a reference because it contains few sympathetic nerves. Therefore, average counts per pixel in the heart and mediastinum are used for calculation. The washout ratio (WOR) of MIBG is another parameter used. It indicates the activity tone of the presynaptic sympathetic nerves, which is significantly higher in PD. The WOR is calculated as follows (33). 

WOR={Heart early-Mediastinumearly-[Heart later-Mediastinum;ater][(Heart early-Mediastinumearly]

Note: early = 15 minutes and late = 4 hours after ^131^I-MIBG administration

 The H/M ratios at early (15 minutes) and late (4 hours) time points and WOR of ^131^I-MIBG are shown in Table 15 (33).

 H/M ratio can also differentiate dementia with Lewy bodies (DLB) and Alzheimer’s disease (AD). 

 Since the Lewy bodies also causes DLB, myocardial uptake is also diminished. A study using ^131^I-MIBG (43) demonstrated that the H/M ratio at early (30 minutes) and late (4 hours) time points were lower in DLB than in AD, as shown in Table 16.

**Table 15:** Early (15 minutes) and late (4 hours) H/M ratio with standard deviation (SD) of 131I-MIBG in HC, ET, MSA, and PD

**Condition**	**Early H/M ratio**	**Late H/M ratio **	**WOR**
HC	2.41 ± 0.26	2.66 ± 0.47	−7.87 ± 32.65
ET	2.34 ± 0.34	2.46 ± 0.51	−4.70 ± 30.75
MSA	1.97 ± 0.36	2.08 ± 0.57	9.96 ± 30.88
PD	1.65 ± 0.36	1.50 ± 0.43	32.58 ± 22.99

Note: HC = healthy control

**Table 16:** Early (30 minutes) and late (4 hours) H/M ratio range of 131I-MIBG in Dementia with Lewy body (DLB) and Alzheimer’s disease (AD)

**Condition**	**Early H/M ratio**	**Late H/M ratio**
DLB	1.37-1.49	1.12-1.54
AD	1.72-1.79	1.78-1.80

***TRODAT-1 brain SPECT***

***General Considerations***

 TRODAT SPECT represents the dopamine transporter. The normal study shows symmetrical radiotracer uptake in both caudate nuclei and putamina with very low or absent background activity. The head of caudate and putamen show the highest activity, as seen in the “comma” shape for all ages. However, the degree of striatal binding may decrease with normal aging by about 5-7% per decade (44). However, the pattern for visual interpretation remains unchanged. TRODAT SPECT imaging is not recommended to distinguish between PD and atypical Parkinsonism and among atypical Parkinsonism.

***Image Data Display***

 TRODAT-1 brain SPECT was displayed in the axial view in gray and color scale using the level of anterior commissure-posterior commissure (AC-PC) line. Raw data images must be checked for movement correction. The regions of interest (ROIs) were bilateral striata, bilateral caudate nuclei, and bilateral putamina.

***Image Interpretation***

**Figure 4:**
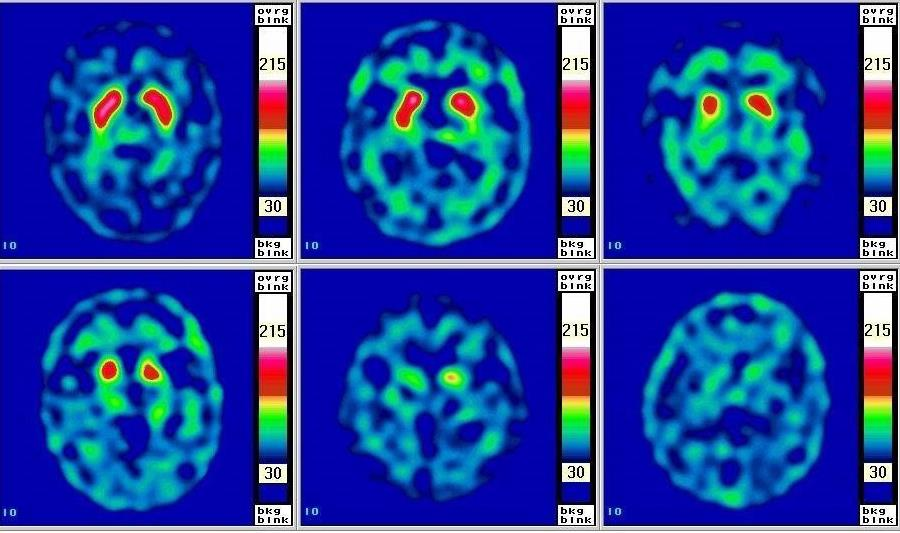
Normal to abnormal TRODAT-1 patterns in H-Y STAGE 0-5

1. Visual Inspection (table 17 and figure 4)

2. Semi-Quantitative Analysis 

**Figure 5:**
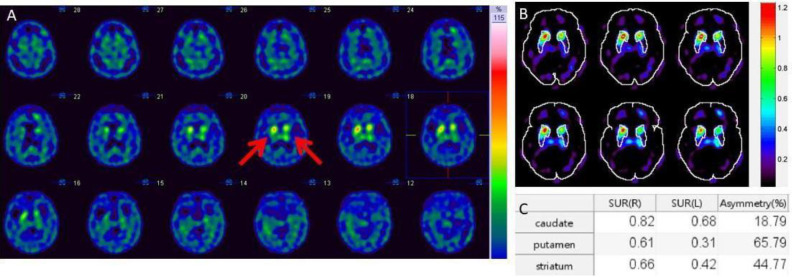
The TRODAT-1 SPECT in Parkinson’s disease with H-Y stage II. **A**: the TRODAT-1 SPECT showed moderately asymmetrical decreased DAT activity in bilateral putamina, and caudate nuclei. **B** and **C** showed a 65.79% asymmetry of putamen uptake. SUR of left putamen is 0.31, while SUR of right putamen is 0.61

 Semi-quantitative analysis helps to determine disease accurately by calculating the specific uptake ratio (SUR) and asymmetrical percentage of bilateral striata (Figure 5). The percentage of asymmetry is significant if more than 10%, indicating early Parkinson’s disease (45). There is no consistently accepted cut-off value for SUR since the value can be influenced by various factors including reconstructed image resolution, attenuation correction, and the size and the placement of regions of interest. The three or more transverse consecutive slices with the highest uptake at striatum were summed up and analyzed. The regions of interest were bilateral striata, bilateral caudate nuclei, and bilateral putamen. The size and shape of ROI should be fixed for each individual.

 $\mathrm{Specific uptake ratio}\left(\mathrm{SUR}\right)=\frac{\left(\mathrm{counts region of interest}-\mathrm{occupital counts}\right)}{\mathrm{occupital counts}}$   (46, 47)

Region of interest = caudate head, putamen, striatum

$\mathrm{Asymmetrical ratio of bilateral striata}=\frac{\left(\mathrm{SUR right striatm}-\mathrm{SUR left striatum}\right)\mathrm{x2x100\%}}{\left(\mathrm{SUR right striatm}+\mathrm{SUR left striatum}\right)}$ 41

**Table 17:** TRODAT-1 Visual Interpretation

**Visual score and findings**		**Possible disease**
**Normal**	Class 0 = normal bilateral uptake of striatum	Normal
		ET
		drug-induced
		parkinsonism
		vascular parkinsonism
		psychogenic
		parkinsonism
		SWEDDs
		AD
**Abnormal**	Class 1 = normal caudate uptake, loss putamen uptake > 50% on one side	Early PD
	Class 2 = normal caudate uptake, loss putamen uptake > 50% both sides	Early PD
	OR decreased caudate and putamen uptake about 50% on both sides	Early PD
	Class 3 = loss caudate uptake > 50% and no putamen uptake	Advanced PD
	Class 4 = finding between class 3 and 5	Advanced PD
	Class 5 = no striatal uptake both sides	Advanced PD

Note: Reduction of TRODAT-1 uptake in the striatum can be found in PD, DLB, atypical Parkinsonism such as PSP, MSA, and CBD

Reprinted by permission from: [Springer] [Eur J Nucl Med Mol Imaging] [Usefulness of brain ^99m^Tc-TRODAT-1 SPET for the evaluation of Parkinson’s disease. Huang.WS, Lee.MS, Lin. JC et al.] [COPYRIGHT] (2004)

***FDG Brain PET***

***General Considerations***

 Brain glucose metabolic rate is considered to be a general marker of neuronal function. Thus, PET/CT imaging by ^18^F-FDG reflects regional neuronal function and provides the disease-specific patterns of resting regional glucose metabolic change accompanying movement disorders. 

***Image Data Display***

1. Axial image in AC-PC (Anterior commissural-Posterior commissural plane) orientation as well as coronal and sagittal views.

2. Image co-registration with MRI image is an option for accurate anatomical localization.

***Image Interpretation***

**Figure 6:**
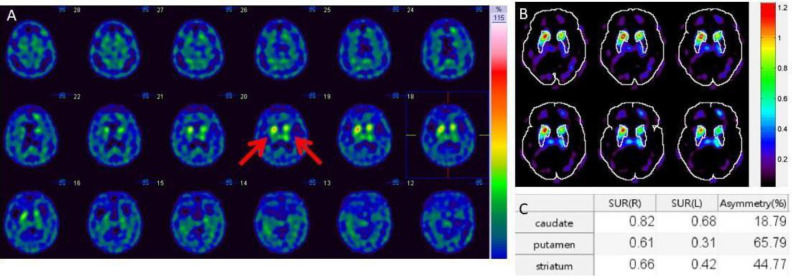
Summarization of brain glucose metabolic change in movement disorders

 Summarized findings of each movement disorder abnormality are shown in Table 18 and Figure 6(2, 48, 53).

**Table 18:** FDG Brain PET Findings in Parkinson’s disease and Parkinsonism

**Disease**	**Findings**
**Parkinson disease**	Increased FDG uptake: thalamus, striatum (or normal), sensorimotor cortex, cerebellum
	Decreased FDG uptake: temporal, parietal, possible occipital, and frontal cortices in demented PD (associated with future cognitive impairment)
**MSA**	Decreased FDG uptake
	MSA-P: striatum
	MSA-C: cerebellum
**PSP**	Decreased FDG uptake: dorsolateral frontal cortex, medial frontal cortex, thalamus, caudate nucleus, and upper brain stem
**CBD**	Decreased FDG uptake: asymmetrical at the striatum, medial, and lateral frontoparietal cortices (contralateral to the most affected body side)
**DLB**	Decreased FDG uptake: parietotemporal association cortices, occipital association cortex, and primary visual cortex (medial occipital lobe), bilaterally. FDG uptake in the posterior cingulate cortex could be relatively less affected

***FDOPA PET***

***General Consideration***

 FDOPA or 6-fluoro-(^18^F)-L-3, 4-dihydroxy-phenyl -alanine is the amino acid analog precursor for dopamine. Therefore, FDOPA can detect abnormal dopaminergic presynaptic activity in movement disorder, confirming the clinical diagnosis of degenerative Parkinsonism (54).

***Image Data Display***

- The serial images would be re-oriented into the AC-PC plane and displayed in the axial plane. 

 The image orientation of symmetrical adjustment should be performed carefully to provide appropriate relative visual analysis to compare the degree of radiopharmaceutical uptake at both sides of the regional brain.

- Generally, the slice selection for image scaling can show the most prominent crescent shape of the putamen and the rounded shape of caudate.

***Image Interpretation***

**Figure 7:**
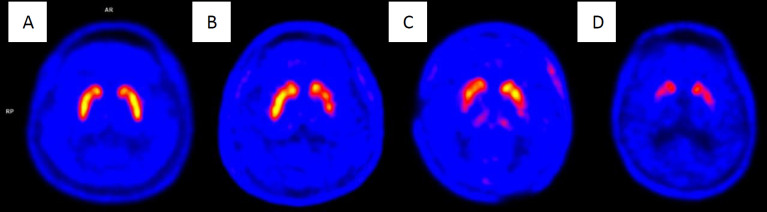
(**A**) A Normal FDOPA PET scan with intense symmetrical activity in bilateral striata. There is various severity of abnormal FDOPA PET scan in movement disorder as seen (**B**) showing asymmetrical decreased FDOPA uptake in left putamen and caudate with normal on the right side, (**C**) showing asymmetrical decreased FDOPA uptake in bilateral putamen and right caudate, and (**D**) showing decreased FDOPA uptake in bilateral caudate and putamen (more on the right side)

 The imaging findings and interpretation are shown in Table 19 and Figure 7.

**Table 19:** FDOPA PET Findings in Movement Disorder

**Interpretation**	**Findings**	**Possible disease**
**Normal scan**	the crescent shape of the putamen and rounded shape of caudate bilaterally symmetric and uniform thickness in both caudate and putamen (comma- or crescent-shaped) Normal ET drug-induced parkinsonism vascular parkinsonism psychogenic parkinsonism SWEDDs	
**Abnormal scan**	reduction in size or thickness and shape of putamen or both putamen and caudate (unilaterally or bilaterally) Early stage: decrease at posterior putamen, contralateral to the affected side of the body Late stage: Decrease at caudate and putamen PD PDD PSP CBD MSA	

***Differential Diagnosis in Parkinsonism***

 FDOPA PET imaging is not recommended to distinguish between PD and atypical Parkinsonism and among atypical Parkinsonism.

 However, it can be used to differentiate between degenerative Parkinsonism and non-parkinsonian disorders, including essential tremor (ET) diagnosis, vascular Parkinsonism, drug-induced Parkinsonism, and other non-parkinsonian disorders (55). Alteration of FDG uptake pattern in the striatum and the cortical brain area can be used to differentiate among degenerative Parkinsonism.

**Figure 8:**
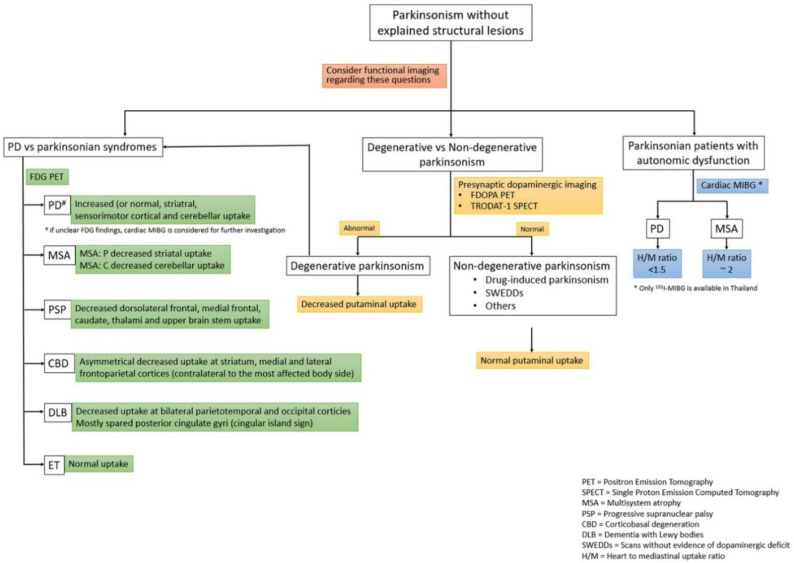
Summarization of nuclear medicine investigation and typical findings in movement Disorder

 Summarization of nuclear medicine investigation and typical findings in movement disorder are shown in Figure 8.

## Reporting Format

 Specific identification of each patient, referring clinician, date and time of the study, and the reporting physician’s signature must be provided in the report as part of quality assurance.

 The recommended structural body of the report includes five major portions as follows:

1. History: The relevant history should be noted, such as specific symptoms and predominant side of movement disorder, the time duration 

from the disease onset, current medication, and withdrawal time interval of drugs interfering with the study.

2. Indication

3. Technique

a. Radiopharmaceutical: type, dosage, and route of administration

b. Detailed imaging and processing techniques should be mentioned, including imaging quality and limitations. If certain specific software or anatomical co-registration is utilized, it should be additionally detailed.

c. Ancillary drugs (if used), e.g., type, route, and time of sedative drugs.

d. Serum glucose level for FDG PET.

4. Findings.

a. Quality of image, e.g., good, fair, poor (e.g., low count rate, motion).

b. Visual analysis, e.g., quality of uptake in caudate and anterior/posterior putamen of each side for TRODAT-1 or ^18^F-FDOPA. A term of hypometabolism and hypermetabolism should be used for ^18^F-FDG. The location, extension, and severity should be reported.

c. Semi-quantitative analysis, e.g., type of normal database comparison used with Z-score cut-off, H/M ratio, and WOR for MIBG, TRODAT-1 SUR of caudate, putamen, and striatum, as well as the percentage of asymmetry should be reported.

 d. Correlative imaging findings, if available, e.g., MRI, CT should be mentioned.

5. Interpretations/Impressions/Conclusions: The final impression from imaging data should be interpreted along with available clinical and correlative imaging data. If they seem discordant, direct discussion with the referring physician is recommended. Also, suggest additional study in some instances to clarify the diagnosis of the current study.

## Pitfall and Error

 Many factors, including biological and technical factors, affect image interpretation, as shown in Table 20(26, 28, 29, 44, 56-67).

**Table 20:** Pitfalls and Errors

**Presynaptic dopaminergic imaging, including TRODAT-1 brain SPECT and FDOPA brain PET**	
**Biological factors**	**Technical factors**
1. Normal variations Decreased striatal binding in normal aging Decreased striatal binding in postmenopausal women Mild asymmetrical striatal uptake 2. Altered location or shape of the striatal structures due to anatomic lesions 3. Medications or drugs that may alter tracer binding (see Table XX in patient preparation section).	Technical factors 1. Artifacts Patient movement Misalignment causing artificial asymmetry 2. Variability of semi-quantitative analysis - Interobserver variability with manual ROI - Correction effects, e.g., attenuation, scatter, and partial volume effect 3. Suboptimal image display plane
**Cardiac MIBG Imaging**	
**Biological factors**	**Technical factors**
1. The progressive decline of MIBG uptake with aging 2. Age dependent metabolic and cardiovascular conditions 3. Heart diseases, e.g., ischemic heart disease, heart failure, dilated cardiomyopathy, myocardial damage, cardiac rhythm disease 4. Autonomic neuropathy or ganglionopathy, e.g., DM, post-polio syndrome 1. Medications False positive (Amiodarone) False-negative (Competitive compounds for NE transporter binding, calcium channel blocker, long-term amiodarone)	1. Artifacts Patient movement 2. Abnormal anatomy causing the erroneous position of mediastinal ROI
**FDG PET**	
**Biological factors**	**Technical factors**
1. Unexpected brain activity from external stimuli 2. Drug interference, e.g., psychotropic drugs, corticosteroid 3. The effects from sedation at the time of injection 4. Anatomical variations 5. Generalized reduced brain FDG uptake secondary to high blood glucose level 6. Recent radio- or chemotherapy 7. Age effect altered brain metabolism	1. Artifacts Patient movement Inappropriate processing, e.g., the reconstruction method 2. Non-continuous color table display 3. Level of contrast and background subtraction 4. Suboptimal image display plane, e.g., not true AC-PC plane, asymmetrical display 5. Partial volume effect on the corrected image

## Statement and declaration

 This guideline has got financial support from Global Medical Solution (GMS) Thailand, Siemens Healthcare, and Premier Business Inter.

 All authors declare that they do not have any commercial or financial relationship that could be construed as the potential conflict of interest.

## Conflict of interest

 None.

## Author’s contributions

 Conceptualization, S.T., B.Kh., T.K.; literature review, all authors; rate recommendation for indication in nuclear medicine imaging, P.L., O.P.; nuclear medicine imaging procedure, radiopharmaceuticals and interpretation, S.T., B.Kh., T.K., W.C., N.W., Ch.C., P.K., P.P., T.S., N.P, S.A.; pitfalls and errors, T.Th.; original draft preparation, T.K, B.Kh., S.T.; gathering external peer review, T.K.; writing review and editing, T.K., S.T.. All authors have read and agreed to the published version of this manuscript.
